# Interaction between intensity and duration of acute exercise on neuronal activity associated with depression-related behavior in rats

**DOI:** 10.1186/s12576-020-00788-5

**Published:** 2021-01-15

**Authors:** Ryoko Morikawa, Natsuko Kubota, Seiichiro Amemiya, Takeshi Nishijima, Ichiro Kita

**Affiliations:** Department of Human Health Sciences, Tokyo Metropolitan University, 1-1 Minami-ohsawa, Hachioji, Tokyo 192-0397 Japan

**Keywords:** Exercise, 5-HT, CRF, Depression, Immunohistochemistry

## Abstract

We examined the activities of serotonin (5-HT) neurons in the dorsal raphe nucleus (DRN) and corticotropin-releasing factor (CRF) neurons in the hypothalamic paraventricular nucleus (PVN) during acute treadmill running at different speeds (control, low, high) and durations (15, 30, 60 min) in male Wistar rats using c-Fos/5-HT or CRF immunohistochemistry. We also performed elevated plus maze test (EPM) and forced swim test (FST) after acute treadmill running in rats. Acute treadmill running at low speed, regardless of exercise duration, significantly increased c-Fos expression in 5-HT neurons in the DRN compared with controls, whereas high-speed running significantly activated 5-HT neurons only at 60-min duration. In contrast, c-Fos expression in CRF neurons in the PVN was enhanced in an intensity-dependent manner, regardless of exercise duration. c-Fos expression in 5-HT neurons in the DRN induced by the acute treadmill running for 30 or 60 min, but not 15 min, was positively correlated with the time spent on the open arms in the EPM and was negatively correlated with the immobility time in the FST. These results suggest an interaction between exercise intensity and duration on the antidepressant effects of acute physical exercise.

## Background

Accumulating evidence suggests that physical exercise can help reduce or prevent the incidence of symptoms of depression, including helplessness and anxiety [1–6]. Monoaminergic hypofunction, including the central serotonergic system, is implicated in the pathophysiology of depression [7–9], and many antidepressant medications enhance the activity of central serotonergic neurotransmission [10, 11]. Several studies have suggested that physical exercise produces antidepressant effects by mediating the activation of serotonin (5-HT) neurons in the dorsal raphe nucleus (DRN) [12–14]. It is known that 5-HT neurons in the DRN widely project to emotion-related brain regions including the amygdala, hippocampus and prefrontal cortex, and are associated with antidepressant properties [7, 8, 15, 16]. On the other hand, corticotropin-releasing factor (CRF) neurons in the hypothalamic paraventricular nucleus (PVN), which play an important role as central activators of integrated stress responses including activation of the hypothalamic–pituitary–adrenal axis, are involved in the manifestation of depressive symptoms [17–19]. Previously, we examined the involvement of 5-HT and CRF neurons in acute exercise-induced changes in depression-related behavior in rats and reported that low-intensity (15 m/min, 30 min), but not high-intensity (25 m/min, 30 min) acute exercise activated DRN 5-HT neurons without producing a high activation of PVN CRF neurons, and thus may effectively improve depression-related behavior [14, 20]. However, we only examined the involvement of exercise intensity in antidepressant effect in 30-min acute exercise, and the effect of a shorter- or longer-acute exercise at different intensities was not considered. Even though several studies have suggested the potential for differential effects of acute exercise duration on the spatial and temporal profiles of neuronal activation in the brain [21–24], to date, few studies have investigated the effects of acute exercise with controlled exercise parameters such as intensity and duration of exercise on antidepressant properties, including behavioral and neural changes. That is, it remains unclear how exercise intensity and duration of acute physical exercise interact with antidepressant effects. Indeed, most previous studies have used diverse exercise regimens, and it has been reported that the beneficial effects of physical exercise vary depending on the exercise parameters, such as the intensity, duration, and type of exercise [5, 25–29]. Therefore, the optimal exercise regimen for the treatment of depressive symptoms remains unclear. Understanding the general concepts regarding acute physical exercise regimens that effectively improve the neuronal activity associated with depression-related behavior may provide a standardized exercise protocol for the treatment of depressive symptoms.

This study represented an extension of our previous reports [14, 20], with inclusion of a shorter (15 min) and longer (60 min) acute exercise at different intensities, as well as different 30-min exercise group from previous reports, and we attempted to examine the effects of a bout of acute exercise at different intensities and durations on the neuronal activity associated with depression-related behavior in rats. To accurately control the intensity and duration of exercise, we adapted forced treadmill running as the exercise protocol. We examined the activities of 5-HT neurons in the DRN and CRF neurons in the PVN during acute treadmill running at different intensities and durations in rats, using double-staining for c-Fos, which is a well-known transcription factor frequently used as a functional marker of neuronal activity [30–33], and 5-HT or CRF. We also performed elevated plus maze test (EPM) and forced swim test (FST) after acute treadmill running, to assess depression-related behavior in rats according to our previous reports [14, 20]. Furthermore, because activation of 5-HT neurons is known to be implicated in antidepressant/anxiolytic properties [7, 8, 10, 11, 34], the relationship between c-Fos expression in 5-HT neurons in the DRN and depression-related behavior was determined for each exercise duration. Our results suggest the existence of an interaction between exercise intensity and duration on neural activity associated with the antidepressant effects of acute physical exercise.

## Methods

### Animals

Seventy-two adult male Wistar rats (weight 240–280 g) were used for the experiments (Sankyo Labo Service Corporation, Tokyo, Japan). All rats were caged in groups of 3–4 under controlled temperature (23 °C) and light conditions (12-h light/12-h dark cycle, lights on at 05:00) with ad libitum access to food and water. All experimental procedures were carried out in accordance with the European Communities Council Directive of November 24, 1986 (86/609/EEC) and were approved by the Animal Experimentation Ethics Committee of Tokyo Metropolitan University. All efforts were made to minimize animal suffering and the number of animals used.

### Experimental procedures

**Fig. 1:**
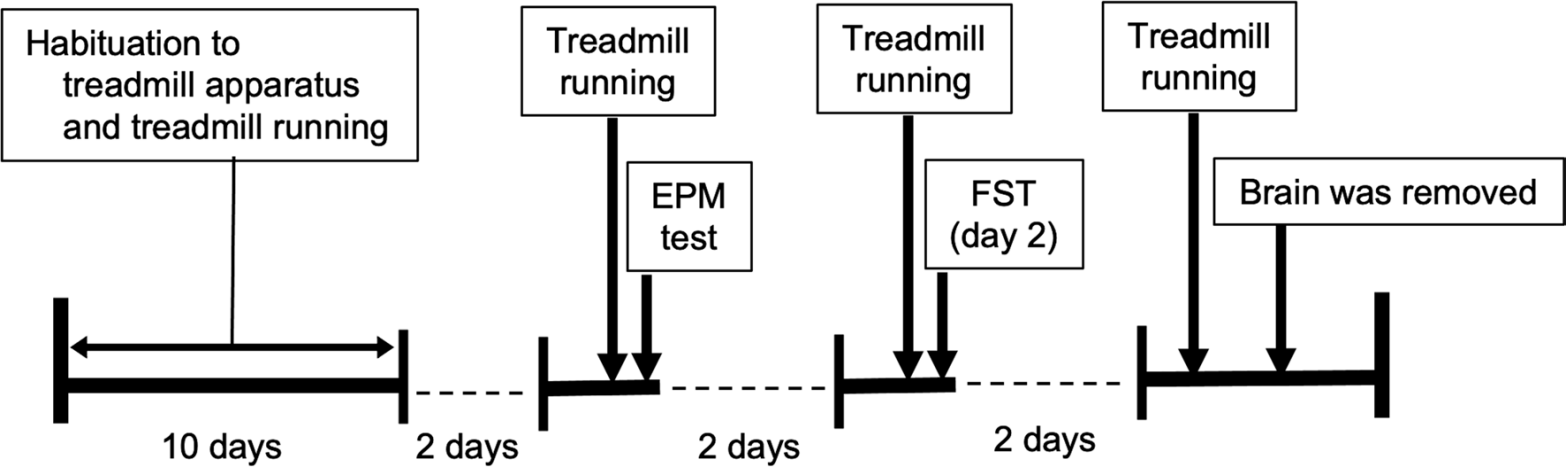
Timeline of the experiment. Rats were habituated to the treadmill apparatus and treadmill running for 10 days. Then, the behavior in elevated plus maze test (EPM) and forced swim test (FST) was evaluated 30 min after a bout of treadmill running (separated by 2 days). Two or more days after behavioral testing, rats performed the same treadmill running again and were sacrificed, and the brains were removed for immunohistochemistry

A schematic of the experimental timeline is presented in Fig. 1. All treadmill procedures were conducted in the dark period of the light/dark cycle. All rats were habituated to the treadmill apparatus and treadmill running for 10 days, in accordance with previous studies [14, 20, 35]. The 10-day running habituation protocol consisted of running on a motor-driven treadmill at 0° incline with incremental increases in speed (from 0 to 25 m/min) and duration (from 15 to 60 min). Rats that were unable to keep up with the pace of the treadmill received a mild but aversive foot shock (0.1 mA) provided by shock grids at the rear of the treadmill to maintain the correct regimen. Very few foot shocks (0–4 shocks/day for each rat) were administrated during the habituation session. In rare cases, two of all rats received excessive numbers of foot shocks or refused to run; these rats were excluded from the experiment, to minimize the potentially confounding effect of foot shocks on treadmill running such as conditioned fear. After the habituation period for all rats, the rats performed a bout of acute treadmill running at 1 of 3 different intensities (SED, sedentary control, 0 m/min; LSR, low-speed running, 15 m/min; or HSR, high-speed running, 25 m/min) and durations (15, 30, or 60 min). Rats in the LSR and HSR groups performed treadmill running without foot shocks. The SED group was placed on the stationary treadmill for 15, 30, or 60 min. LSR and HSR corresponded to the sub- and supra-lactate threshold (LT), respectively, because the LT in rats is at a running speed of approximately 20 m/min [35, 36]. Thirty minutes after each bout of treadmill running, at which the levels of blood lactate can almost recover to a level before the running [36, 37], all rats performed two types of behavioral tests, the elevated plus maze test (EPM) and the forced swim test (FST) (separated by 2 days), to evaluate depression-related behavior, including helplessness and anxiety. At 2 days or more after behavioral testing, rats performed the same treadmill running again and were sacrificed, and the brains were removed for immunohistochemistry.

### Behavioral tests

*EPM:* We performed EPM after acute exercise according to our previous study [14]. The elevated plus maze comprises two open arms (50 cm long, 10 cm wide, 0.5 cm rim around the edges) and two closed arms (50 cm long, 10 cm wide, 45 cm high wall) extending from a common central platform (10 × 10 cm). The apparatus was positioned 50 cm above floor. Each rat was individually placed in the central platform facing an open arm after acute exercise, and allowed to explore the maze for 5 min. The time spent on the open arms, the frequency of entries into the open arms and the frequency of total arm entries were monitored via a video camera mounted above the maze as measures of anxiety-like behavior and locomotor activity.

*FST:* The FST was performed according to methods previously described [14, 20, 38]. The FST consists of an initial 15-min swim session on day 1 and a 6-min swim test at 24 h after the session on day 1. Each rat was individually placed in a plexiglas™ cylinder (25 cm diameter, 50 cm height) containing water (35 cm deep, 23–25 °C) and monitored via a video camera for 6 min on day 2. The immobility time during the last 5 min of the 6-min swim test was scored by an observer as an index of helplessness or behavioral despair. The observer was blinded to the type of exercise. Immobility was defined as the absence of movement but included the presence of movements necessary to keep the head above water.

### Immunohistochemistry

To assess the neuronal activities during acute treadmill running at different intensities and durations in rats, we used double-staining for c-Fos and 5-HT or CRF. Eighty minutes after the end of each bout of treadmill running, rats were deeply anesthetized using an intraperitoneal injection of sodium pentobarbital (50 mg/kg) and perfused transcardially with heparin solution (1000 U/l, 0.9% saline), followed by ice-cooled 4% paraformaldehyde, 0.1% glutaraldehyde and 0.2% picric acid in 0.1 M phosphate-buffered saline (PBS; pH 7.4). The rat brains were removed and post-fixed in the same fixative without glutaraldehyde for 24 h at 4 °C. The brains were then cryoprotected in a phosphate-buffered 30% sucrose solution with 0.1% sodium azide for 24–48 h. Next, the brains were frozen and cut in the coronal plane (6 series of 40-µm thick sections) on a microtome and collected in 0.1 M PBS with 0.1% sodium azide.

Two of 6 series of sections were selected for immunohistochemistry for c-Fos and 5-HT or CRF. Immunohistochemical visualization of c-Fos and 5-HT or CRF was carried out on free-floating sections using antibodies and avidin–biotin-peroxidase methods, as described previously [14, 29, 39–41]. Briefly, after blocking endogenous peroxidase and pre-incubation in 10% normal horse serum, the brain sections were incubated in primary rabbit polyclonal anti-Fos antiserum (1:600, sc–52; Santa Cruz Biotechnology, Santa Cruz, CA, USA) diluted in 0.1 M PBS with 0.1% Triton X-100 (PBS-TX) for 16 h at room temperature. After rinsing 3 times for 5 min in PBS-TX, the sections were further incubated in a biotinylated secondary antibody solution, donkey anti-rabbit IgG (1:800, AP182B; Chemicon, Temecula, CA, USA) for 90 min at room temperature, rinsed 3 times for 5 min in PBS-TX, and finally treated with the avidin–biotin-peroxidase complex (1:400, Vectastain ABC peroxidase kit; Vector Laboratories, Burlingame, CA, USA) for 90 min. The sections were reacted for peroxidase activity in a solution of nickel ammonium sulfate, 0.02% 3,3′-diaminobenzidene (DAB) in 0.1 M Tris–HCl buffer (pH 7.6) and 0.01% H_2_O_2_ for 20 min. Immunoreactivity for c-Fos was localized to cell nuclei, appearing as a dark gray-black stain. No staining was observed on sections incubated without the primary antibodies (sc-52). For dual immunostaining for 5-HT or CRF, the same sections were sequentially incubated in 5-HT antibody (1:5000, 20079; Immunostar Inc., Hudson, WI, USA) or CRF antibody (1:5000, T-4037; Peninsula Laboratories, San Carlos, CA, USA). Avidin–biotin-peroxidase complexes were visualized using DAB in 0.1 M Tris–HCl buffer without nickel sulfate. 5-HT or CRF immunoreactivity was localized to the cell cytoplasm and was visible as light-brown staining. No staining was observed on sections incubated without the primary antibodies (20079 for 5-HT or T-4037 for CRF). Sections were then washed in 0.1 M PBS, mounted onto gelatin-coated slides, air-dried, dehydrated in graded alcohol, cleared in xylene, and coverslipped with Permount mounting medium (Fisher Scientific, Pittsburgh, PA, USA).

### Cell counts and quantification

Cell counts and quantification were performed as previously described [14, 20]. Immunoreactive cells on sections were observed using an optical microscope (BX-50; Olympus, Tokyo, Japan) equipped with a charged-coupled device camera (DP-73; Olympus). For unbiased quantification, slides were coded prior to analysis. Quantitative analysis was performed on sections containing the DRN and PVN. The total numbers of 5-HT-positive and double-labeled cells for c-Fos and 5-HT in the entire DRN were manually counted on sections between –7.3 and –8.3 from the bregma (7–8 sections; corresponding to Plates 94–102 in the *Paxinos and Watson The Rat Brain in Stereotaxic Coordinates* [42]), and the percentage of c-Fos-positive 5-HT neurons per section was calculated. Similarly, the total numbers of CRF-positive and double-labeled cells for c-Fos and CRF in the PVN were counted bilaterally on sections between − 1.3 to − 2.0 from the bregma (3–4 sections; corresponding to Plates 44–50 in the *Paxinos and Watson The Rat Brain in Stereotaxic Coordinates* [42]), and the percentage of c-Fos-positive CRF neurons per section was calculated.

### Statistical analysis

Values for behavioral data and immunoreactive cells are expressed as the mean ± standard error of the means. Statistical evaluations of the experiments were performed using two-way analysis of variance (ANOVA; i.e., 3 intensities [SED, LSR, HSR] × 3 durations [15, 30, 60 min]), followed by Scheffe’s post-hoc analysis. Effect size (ES) in simple main effect test (i.e., one-way ANOVA) for intensity was calculated for each exercise duration using partial eta-squared to estimate the magnitude of effect of exercise intensity on EPM and FST. The correlations between the c-Fos-positive 5-HT neurons and depression-related behavior, i.e., the time spent on the open arms in the EPM and the immobility time in the FST, were analyzed for each exercise duration, as well as for combined data of all durations, using Pearson’s correlation coefficient. All analyses were performed using SPSS (version 25.0; IBM, Tokyo, Japan). Values of *p* < 0.05 were considered statistically significant.

## Results

### Effects of acute treadmill running on neuronal activities

**Fig. 2:**
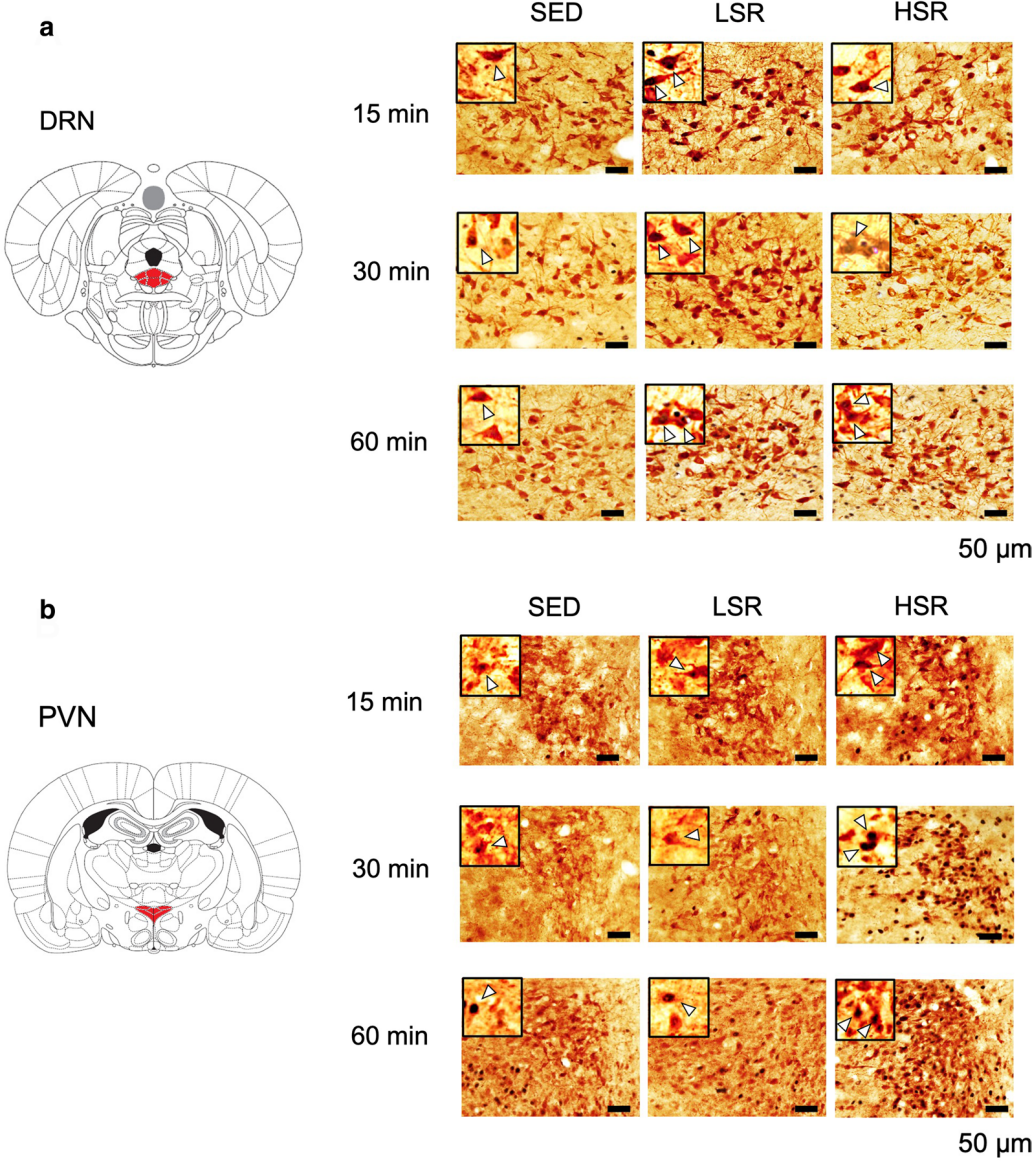
Photographs of double-staining for c-Fos and serotonin (5-HT) in the dorsal raphe nucleus (DRN; panel **a**) or corticotropin-releasing factor (CRF) neurons in the hypothalamic paraventricular nucleus (PVN; panel **b**) of rats after acute treadmill running. SED, sedentary controls; LSR, low-speed running; HSR, high-speed running. Arrowheads indicate double-labeled cells. Scale bars are 50 µm

**Fig. 3:**
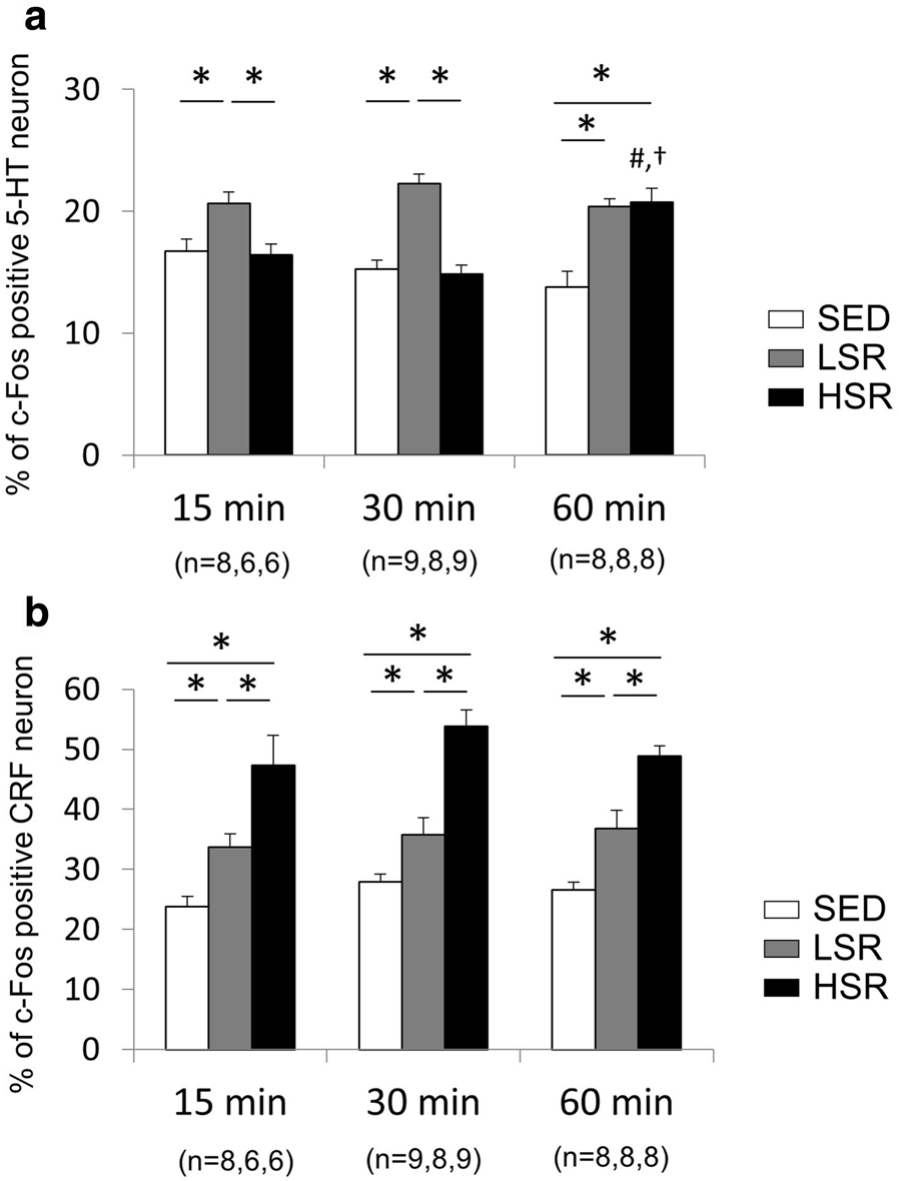
Mean (± standard error of the mean) percentage of double-labeled cells in the dorsal raphe nucleus (**a**) and the hypothalamic paraventricular nucleus (**b**) of rats during acute treadmill running. *SED* sedentary controls, *LSR* low-speed running, *HSR* high-speed running, *5-HT* serotonin, *CRF* corticotropin-releasing factor. *p < 0.05 vs. the other group for each duration of exercise; ^#^*p* < 0.05 vs. HSR for 15 min; ^†^*p* < 0.05 vs. HSR for 30 min

We performed double-staining for c-Fos and 5-HT or CRF after a bout of acute treadmill running (Fig. 2). Results revealed that acute treadmill running at a low speed increased c-Fos expression in 5-HT neurons in the DRN regardless of the exercise duration, whereas high-speed treadmill running increased c-Fos expression in 5-HT neurons in the DRN only with a 60-min duration (Fig. 3a). Two-way ANOVA revealed a significant main effect for exercise intensity on the percentage of c-Fos-positive 5-HT neurons in the DRN [F(2,61) = 30.059, *p* < 0.01]. In addition, a significant interaction was observed between the intensity and duration of exercise [F(4,61) = 7.254, *p* < 0.01]. Post-hoc analysis revealed that c-Fos expression of DRN 5-HT neurons in the LSR rats was significantly enhanced compared with that in the SED rats, regardless of the exercise duration. In addition, c-Fos expression of DRN 5-HT neurons in the HSR rats performing a 60-min bout of treadmill running was significantly increased compared with that in the SED rats, and was significantly higher than that in the HSR rats with a 15- or 30-min exercise duration. On the other hand, acute treadmill running increased c-Fos expression in CRF neurons in the PVN in an intensity-dependent manner, regardless of the exercise duration (Fig. 3b). Two-way ANOVA revealed a significant main effect for exercise intensity on the percentage of c-Fos-positive CRF neurons in the PVN [F(2,61) = 70.228, *p* < 0.01]; however, no significant interaction was observed. The post-hoc analysis indicated that the percentage of c-Fos-positive CRF neurons in the HSR rats was significantly higher than those in the SED and LSR rats. In addition, the percentage of c-Fos-positive CRF neurons in the LSR rats was significantly higher than that in the SED rats.

### Effect of acute treadmill running on EPM performance

**Fig. 4:**
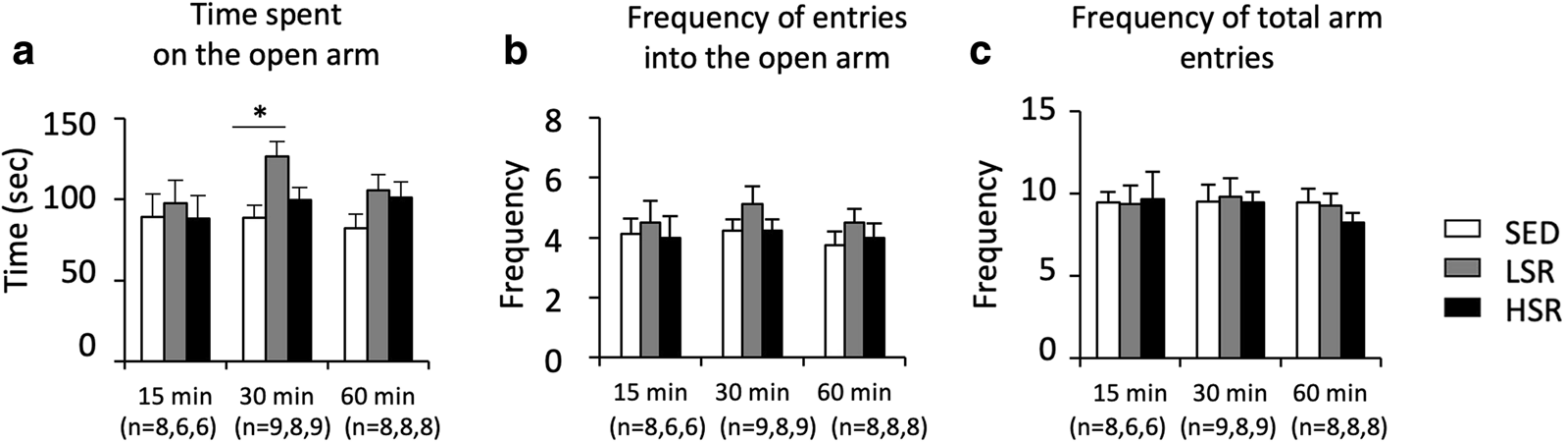
Mean (± standard error of the mean) values in time spent on open arms (**a**), frequency of entries into the open arms (**b**), and frequency of total arm entries (**c**) in the elevated plus maze test after treadmill running. SED, sedentary controls; LSR, low-speed running; HSR, high-speed running. *p < 0.05 vs. SED group

In the EPM, the LSR rats showed longer time spent on the open arms and slightly increased frequency of entries into the open arms, whereas frequency of total arm entries was less affected by exercise condition (Fig. 4). Two-way ANOVA (intensity × duration) revealed a significant main effect for exercise intensity, but not for duration, on the time spent on the open arms [F(2,61) = 4.335, *p* < 0.05], and no significant interaction was observed. The post-hoc analysis showed that the time spent on the open arms in the LSR rats was significantly longer than that in the SED rats (ES = 0.026, 0.321, 0.143 for 15-, 30-, 60-min duration, respectively). In contrast, two-way ANOVA (intensity × duration) revealed no significant main effects for exercise intensity and duration on the frequency of entries into the open arms and the frequency of total arm entries, and no significant interaction was observed.

### Effect of acute treadmill running on FST performance

**Fig. 5:**
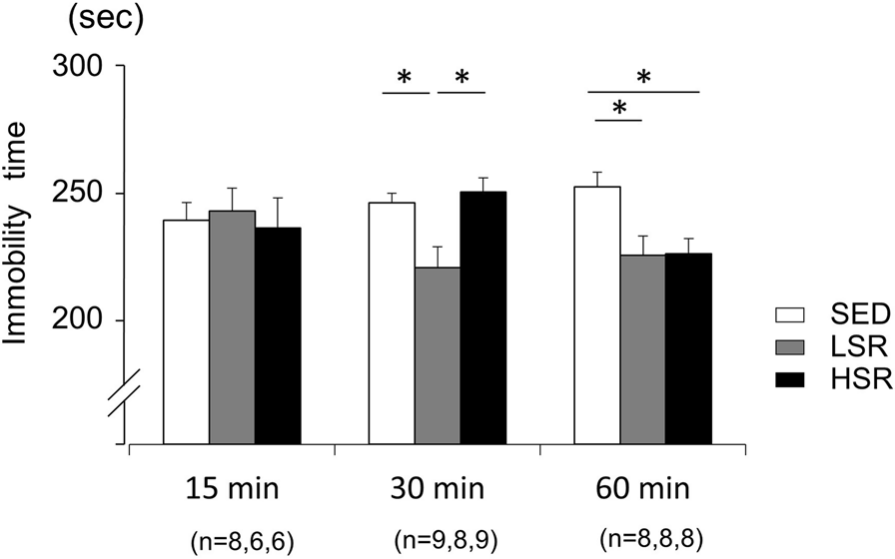
Mean (± standard error of the mean) immobility time in the forced swim test after treadmill running. *SED* sedentary controls, *LSR* low-speed running, *HSR* high-speed running. *p < 0.05 vs. the other group for each duration of exercise

The immobility time in FST, which has been used as a measure of helplessness or behavioral despair [43, 44], was measured 30 min after acute treadmill running in rats (Fig. 5). The immobility time was decreased in the LSR rats performing a 30- or 60-min bout of treadmill running compared with the SED rats. In addition, a 60-min bout of treadmill running at high speed also decreased the immobility time compared with the SED rats. In contrast, a 15-min bout of treadmill running did not alter the immobility time compared with SED rats. Two-way ANOVA revealed a significant main effect for exercise intensity on immobility time [F(2,61) = 3.988, *p* < 0.05]. In addition, a significant interaction (intensity × duration) was observed [F(4,61) = 3.058, *p* < 0.05]. The simple main effect tests indicated significant main effects for exercise intensity on 30- and 60-min treadmill running [F(2,23) = 7.225, *p* < 0.01, ES = 0.386; F(2,21) = 5.404, *p* < 0.05, ES = 0.340, respectively], but not on 15-min treadmill running [F(2,17) = 0.121, *p* > 0.05, ES = 0.014]. The post-hoc analysis showed that the immobility time in the LSR rats performing a 30-min bout of treadmill running was significantly shorter than that in the SED and HSR rats. In addition, the immobility times in the LSR and HSR rats with 60-min duration were significantly shorter than that in the SED rats. In a 15-min bout of treadmill running, no significant difference was observed in the immobility time among the different running speeds.

### Relationships between the c-Fos expression in 5-HT neurons in the DRN and depression-related behaviors

**Fig. 6:**
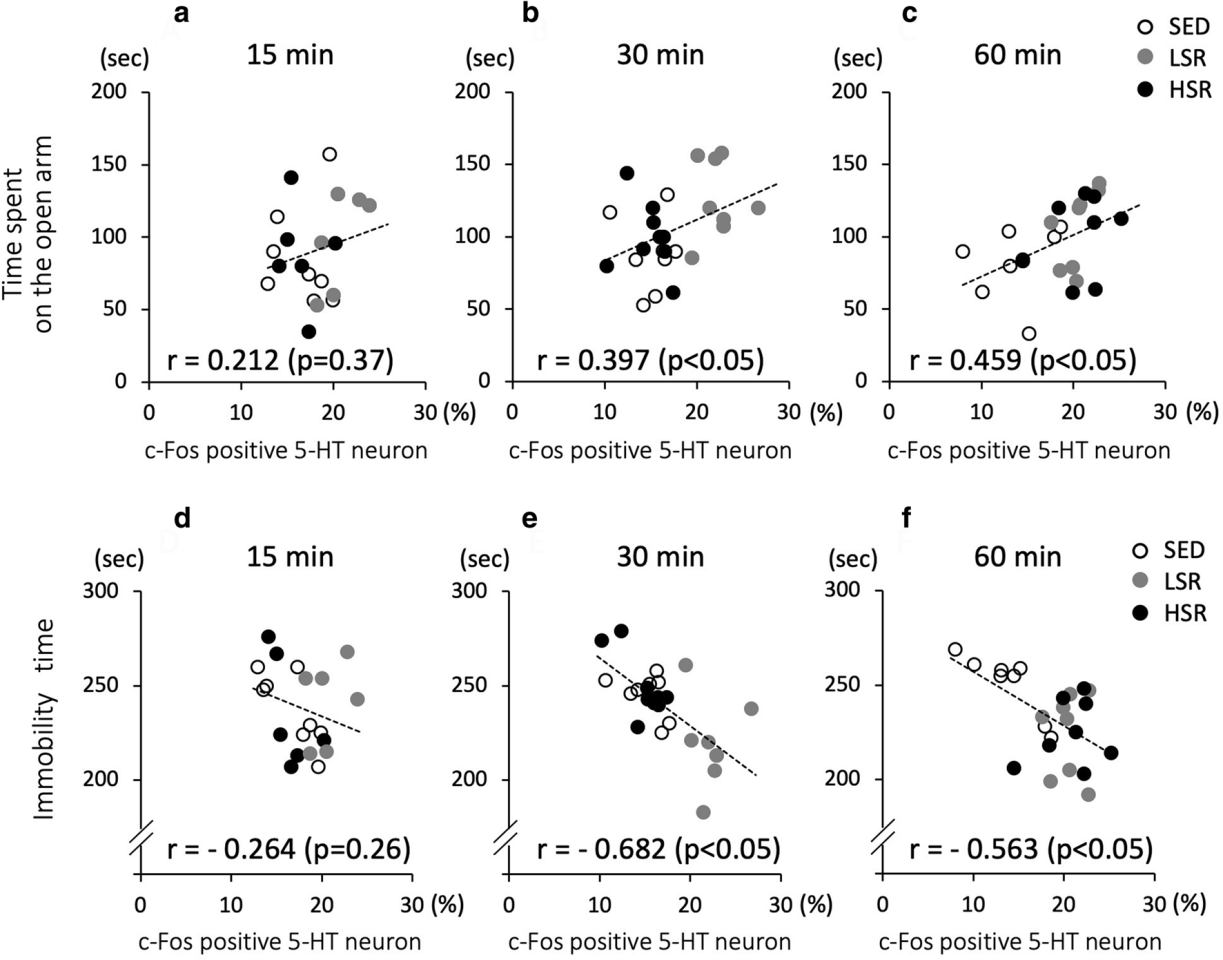
Correlations between c-Fos expression in serotonin (5-HT) neurons in the dorsal raphe nucleus and depression-related behaviors, i.e., time spent on the open arms in the elevated plus maze test (**a**–**c**) and immobility time in the forced swim test (**d**–**f**), for each duration of acute treadmill running. *SED* sedentary controls, *LSR* low-speed running, *HSR* high-speed running. Pearson’s correlations (*r*) are shown in the figures

Pearson’s correlations were performed to determine whether the activity of 5HT neurons in the DRN during acute exercise was involved in the time spent on the open arms in the EPM and the immobility time in the FST. Results showed that c-Fos expression in 5-HT neurons in the DRN was significantly correlated with behavioral responses in combined data of 15-, 30- and 60-min treadmill running (*r* = 0.341, *p* < 0.01 for the time spent on the open arms in the EPM; *r* = − 0.538, *p* < 0.01 for the immobility time in the FST). In addition, for each duration, c-Fos expression in 5-HT neurons in the DRN induced by the acute treadmill running for 30 or 60 min was significantly and positively correlated with the time spent on the open arms in the EPM (Fig. 6b and c) and was negatively correlated with the immobility time in the FST (Fig. 6e and f). In contrast, no significant correlation was observed with a 15-min bout of treadmill running (Fig. 6a and d).

## Discussion

Results of the present study revealed that acute exercise could modulate the neuronal activity associated with depressive symptoms depending on the exercise-condition. More c-Fos-positive 5-HT neurons in the DRN were expressed with low-speed running at durations of 15, 30, and 60 min, whereas a 60-min bout of treadmill running even at high speed enhanced c-Fos expression of DRN 5-HT neurons. In addition, running at high speed enhanced c-Fos expression in CRF neurons in the PVN compared with the control and low-speed running conditions, regardless of the duration. In the EPM, the time spent on the open arms in rats performing low-speed running was longer than that in the control. Furthermore, a 30-min or longer bout of low-speed treadmill running, or a 60-min bout of high-speed running, resulted in decreased immobility time in the FST compared with the controls. Although these results are purely correlative, and therefore, no causality between neuronal activity and behavioral change can be established, the results of this study suggest that a 30-min or longer bout of low-intensity exercise activates 5-HT neurons in the DRN without producing high activation of CRF neurons in the PVN, and may effectively induce antidepressant properties.

Several studies have suggested that acute physical exercise activates the central serotonergic system depending on intensity or duration [14, 21, 23, 45, 46]. For example, Meeusen et al.[23], in a microdialysis study, reported that acute treadmill running at a low speed (12 m/min) for 60 min increased extracellular 5-HT levels in the hippocampus of food-deprived rats in a time-dependent manner from the first 20 min after the onset of running. Similarly, Chaouloff et al. [45] reported that acute treadmill running for 1 or 2 h at 20 m/min (a speed considered low intensity) enhanced brain 5-HT turnover in a time-dependent manner. Previously, using immunohistochemistry, we also examined the effects of a 30-min bout of acute treadmill running at different speeds on the activity of 5-HT neurons in rats and found that a 30-min bout of low-speed treadmill running (15 m/min), but not high-speed treadmill running (25 m/min), enhanced c-Fos expression in 5-HT neurons in the DRN [14]. These findings indicate that mild exercise (low-speed running), even at a shorter duration (< 30 min), efficiently induces the activation of 5-HT neurons in the DRN. In contrast, using in vivo microdialysis, Bequet et al. [47] reported that extracellular 5-HT levels in the rat hippocampus were increased during acute intensive treadmill running (25 m/min, 2 h) from the first 60 min, but not at 30 min after the onset of running, suggesting that intensive exercise may induce a delayed increase in the activation of 5-HT neurons. In the present study, we showed that a bout of low-speed treadmill running at all durations (15, 30, and 60 min), or a 60-min bout of high-speed treadmill running, activated 5-HT neurons in the DRN. Taken together, it is suggested that an interaction exists between exercise intensity and duration on the activation of the central serotonergic system produced by acute physical exercise. In other words, it is possible that although the activation of 5-HT neurons in the DRN can be efficiently induced by low-intensity exercise, a longer-acute exercise even at moderate-to-high intensity may enhance the activity of DRN 5-HT neurons. However, limitation of this study is the fact that all data in immunohistochemistry have been generated from animals which have been exposed to 3 rounds of acute treadmill running with behavioral testing session in between. Thus, the potential confound of those 3 round of acute treadmill running remain to be mitigated.

Although the mechanisms underlying the intensity- and time-dependent response in the serotonergic system activated by acute exercise remain unclear, the magnitude of the stress state during acute exercise may be involved in activation of serotonergic neurons. It has been suggested that the central serotonergic system is controlled by stress-related hormones, including CRF and glucocorticoids [48–50]. In addition, CRF can either increase or decrease serotonergic neuronal activities depending on the concentration and the specific CRF receptor subtype [15, 50]. In the present study, we showed that c-Fos expression in CRF neurons in the PVN during acute treadmill running was increased in a running speed-dependent manner, regardless of the duration, indicating that the intensity and duration of acute physical exercise do not interact with activation of PVN CRF neurons. This suggest that treadmill running at a high intensity is a strong stressor, whereas low-speed running is a milder stressor. Our results indicated that low-speed running could activate 5-HT neurons in the DRN, regardless of the duration. Thus, a high activation of PVN CRF neurons produced by high-intensity acute exercise may have inhibited the effect of acute exercise on activation of DRN 5-HT neurons. Alternatively, a mild activation of CRF neurons may have activated 5-HT neurons. Interestingly, high-speed treadmill running for 60 min also activated DRN 5-HT neurons, despite higher activity of CRF neurons. Thus, the association between 5-HT and CRF neuronal activities during acute exercise may be depended on exercise duration. It suggests the possibility that the neuronal activity of CRF neurons during acute exercise may be involved in the mechanisms underlying the intensity- and time-dependent response in the serotonergic system activated by acute exercise. However, the cellular mechanisms underlying the interaction between CRF and 5-HT systems during physical exercise remain unclear. Further research using methods which manipulate the target neural activity, such as optogenetic approaches, is required to elucidate the mechanisms underlying the intensity- and time-dependent response in the serotonergic system activated by acute exercise.

In recent years, a growing body of literature has examined the influence of a bout of acute exercise on brain function, including affective, mood, and emotional states [1, 51–54]. Most clinical studies examining the effects of acute exercise on affect and mood have suggested that a bout of low-to-moderate exercise can improve psychological well-being [53, 55, 56]. Our results indicated that a 30-min or longer bout of low-speed treadmill running, or a 60-min bout of high-speed treadmill running, decreased the immobility time in the FST compared with controls, suggesting that low-intensity and/or prolonged acute exercise may have positive effects on helplessness or behavioral despair. In addition, a low-speed treadmill running induced longer time spent on the open arms in the EPM compared with the control. Interestingly, our results showed that these exercise conditions could significantly activate 5-HT neurons in the DRN. Therefore, the activation of the serotonergic system during acute exercise may be one of the factors involved in the improvement of depression-related behavior after acute exercise. This scenario is supported by significant correlations between the c-Fos expression in 5-HT neurons in the DRN and the exercise-induced changes in the depression-related behaviors. However, it should be noted that low-speed treadmill running for 15 min did not alter the immobility time, despite the increased activity of 5-HT neurons, and that the correlation between the immobility time and c-Fos expression of DRN 5-HT neurons with a 15-min bout of treadmill running was not significant. This may be somewhat counterintuitive to the results obtained after 30- and 60-min treadmill running. As one explanation, because we conducted a behavioral test at 30 min after the end of a bout of treadmill running, increased brain 5-HT levels induced by a shorter bout of acute low-intensity exercise might have returned to baseline levels within the first 30 min of the recovery period, and thereby, may not have produced a change in behavior. Previous studies [21, 23] have suggested the possibility that the intensity and duration of exercise can affect 5-HT levels in some brain regions not only during exercise, but also during the recovery period. However, the functional relationship between altered c-Fos expression in serotonergic neurons and the behavioral effect is not clear in the present study. Further investigations are needed to clarify the role of the serotonergic system in depression-related behavior after acute exercise, using optogenetic or neuropharmacological approaches.

One limitation of this study is the use of the FST as a method of measure of helplessness or behavioral despair, i.e., a depression-related behavior. Although the FST has been widely used to assess a depression-like phenotype in rodents [43, 44], the rationale for the use of the immobility time in the FST as a measure of depressive-like behavior is not universally accepted. Recently, various alternate hypotheses have been proposed, including stress-coping strategy, stress coping and adaptation, and even anxiety-like behavior [57–59]. Thus, our results of immobility time in the FST may indicate that acute exercise is involved in stress adaptation and alteration in stress coping strategies in an exercise condition-dependent manner. Taken together, we cannot rule out the possibility that the results obtained in this study may provide an alternative interpretation on effects of acute exercise on the immobility time in the FST, not antidepressant effects of acute exercise. What immobility behavior in the FST means exactly remain to be considered. Complementary approaches, including sucrose preference and inescapable foot shock tests are required to gain a complete understanding of the effects of acute exercise on depression-related behavior.

## Conclusions

In conclusion, our results showed that a 30-min or longer bout of acute low-speed treadmill running or a 60-min bout of acute high-speed treadmill running could reduce immobility time in the FST and that a bout of acute low-speed treadmill running could induce longer time spent on the open arms in the EPM, accompanied by the activation of 5-HT neurons in the DRN. Although this study is purely correlative, and therefore, no causality can be established, these results suggest that the effects of acute exercise on the neuronal activation and behavior associated with depression symptoms depend on the intensity and duration of exercise. As long-term adaptation to regular exercise could be the result of the cumulative effects of each type of acute exercise, understanding the interaction between exercise intensity and duration on neural alterations produced by acute exercise may provide new insight into the establishment of optimal exercise regimens to treat depressive symptoms.

## Data Availability

The data that support the findings of this study are available from the corresponding author on reasonable request.

## Abbreviations

CRF: Corticotropin-releasing factor
5-HT: Serotonin
DRN: The dorsal raphe nucleus
PVN: The hypothalamic paraventricular nucleus
EPM: Elevated plus maze test
FST: Forced swim test
SED: Sedentary control
LSR: Low-speed running
HSR: High-speed running
LT: Lactate threshold
